# Track-Density Ratio Mapping With Fiber Types in the Cerebral Cortex Using Diffusion-Weighted MRI

**DOI:** 10.3389/fnana.2021.715571

**Published:** 2021-07-23

**Authors:** Sang-Han Choi, Gangwon Jeong, Young-Eun Hwang, Yong-Bo Kim, Haigun Lee, Zang-Hee Cho

**Affiliations:** ^1^Neuroscience Convergence Center, Korea University, Seoul, South Korea; ^2^AICT, Seoul National University, Seoul, South Korea; ^3^Neuroscience Research Institute, Gachon University, Incheon, South Korea; ^4^Green Manufacturing Research Center, Korea University, Seoul, South Korea

**Keywords:** nerve fibers, brain mapping, diffusion-weighted MRI, human connectome project, tractography

## Abstract

The nerve fibers are divided into three categories: projection, commissural, and association fibers. This study demonstrated a novel cortical mapping method based on these three fiber categories using MR tractography data. The MR fiber-track data were extracted using the diffusion-weighted 3T-MRI data from 19 individuals’ Human Connectome Project dataset. Anatomical MR images in each dataset were parcellated using FreeSurfer software and Brainnetome atlas. The 5 million extracted tracks per subject by MRtrix software were classified based on the basic cortical structure (cortical area in the left and right hemisphere, subcortical area), after the tracks validation procedure. The number of terminals for each categorized track per unit-sized cortical area (1 mm^3^) was defined as the track-density in that cortical area. Track-density ratio mapping with fiber types was achieved by mapping the density-dependent color intensity for each categorized tracks with a different primary color. The mapping results showed a highly localized, unique density ratio map determined by fiber types. Furthermore, the quantitative group data analysis based on the parcellation information revealed that the majority of nerve fibers in the brain are association fibers, particularly in temporal, inferior parietal, and occipital lobes, while the projection and commissural fibers were mainly located in the superior part of the brain. Hemispheric asymmetries in the fiber density were also observed, such as long association fiber in the Broca’s and Wernicke’s areas. We believe this new dimensional brain mapping information allows us to further understand brain anatomy, function.

## Introduction

Neurons in the cerebral cortex are connected with three major brain regions, subcortical, inter-cortical, and intra-cortical regions. Depending on these connected brain regions, the nerve fibers are broadly divided into three categories, i.e., projection, commissural, and association fibers. Projection fibers, also called corticofugal fibers, are connected with numerous subcortical structures in the telencephalon, diencephalon, brain stem, and spinal cord *via* the internal capsule. Association fibers are intra-cortical fibers that connect with cortical regions in the ipsilateral hemisphere, either nearby (short association) or at a distance (long association). Commissural fibers are inter-cortical connections fibers connected with cortical regions in the contralateral hemisphere *via* the corpus callosum and anterior commissure (Afifi and Bergman, 1998; Anthony et al., 2002; David and Anil, 2010).

Briefly, the trajectory for each categorized fiber is clearly distinguished; for example, projection fibers, association fibers, and commissural fibers in the left hemisphere (LH) are connected with the subcortical area, LH, and right hemisphere (RH), respectively. Therefore, it can be presumed that the density of each categorized fiber in each cortical region is also distinguishable. Furthermore, a novel brain mapping method can be considered based on the track-density distribution characteristic for each categorized fiber in the cerebral cortex using brain imaging data, such as the diffusion-weighted MR-based tractography data (Basser et al., 1994, 2000).

The tractography data from the diffusion-weighted imaging (DWI) has been applied for lots of research topics for the human brain, such as analyzing the pattern of the brain connectivity and connectome (Gong et al., 2009; van den Heuvel and Sporns, 2011; Irimia et al., 2012), composing the whole-brain of connectivity-based-parcellation (Fan et al., 2016; Glasser et al., 2016a), clinical studies (Yamada et al., 2009), brain atlas (Oishi et al., 2011; Cho et al., 2015a), and nerve fiber structure (Cho et al., 2015b; Choi et al., 2019, 2020). There are also several studies that applied the tractography data for brain mapping (Behrens et al., 2003; Tomassini et al., 2007; Park et al., 2008; Mars et al., 2011; Liu et al., 2016; Cerliani et al., 2017). Unlike the study for the connectivity-based-parcellation using the tractography data (Fan et al., 2016; Glasser et al., 2016a), the study for the tractography data-based brain mapping does not utilize the detailed brain parcellation information, or the results are not directly associated with the preceded brain parcellation information.

However, most of the previous tractography data-based cortical mapping methods only considered a limited group of nerve fibers in the brain, not whole-brain tractography data. The nerve fibers in the previous tractography-based mapping studies were only associated with a specific brain region, such as the premotor cortex (Tomassini et al., 2007), parietal cortex (Mars et al., 2011), and frontal cortex (Cerliani et al., 2017). Even if not, the nerve fibers in these mapping methods were only interested in particular fiber types, such as the projection fibers from the thalamus for the thalamus segmentation (Behrens et al., 2003). Or they were only considered in commissural fibers for the segmentation of the corpus callosum (Park et al., 2008) or association fibers for the characterization of the cerebral cortex (Liu et al., 2016). Even in the case of cortical mapping that considers the entire nerve fibers, only one-dimensional information was regarded, such as the fiber length (Bajada et al., 2019).

These kinds of brain mapping approaches have enabled a precise understanding of connectivity and function in specific brain regions or brain functions. However, it remains challenging to gain general information of whole-brain states or function through this limited particular fiber connectivity information or single-dimensional information. For understanding the overall aspect of the whole-brain states and functions, there is a need for a mapping approach in which various types of fibers are reflected in multiple dimensions. Therefore, in this report, we demonstrated a multiple- dimensional cortical mapping using a non-specific whole-brain MR fiber-tracking based on the three types of the nerve fiber. Furthermore, the quantitative analysis of the track-density information was also performed.

## Materials and Methods

DWI and anatomical T1-weighted MR imaging data from 3.0T MRI (University of Minnesota, Siemens Skyra 3T) were used in this study. The dataset was obtained from the Human Connectome Project (HCP; Glasser et al., 2016b); 19 individual MRI datasets were selected without any criteria or prior knowledge about subjects (we did not perform any experiment to obtain additional data). The HCP dataset is freely downloadable after a verification process from https://db.humanconnectome.org/app/template/SubjectDashboard.vm?project∼=∼HCP_1200&subjectGroupName∼=∼Subjects%20with%207T%20MR%20Session%20Data. The data contain preprocessed diffusion data with the HCP diffusion pipeline, including diffusion weighting, direction, time series, brain mask, a file that can be used to account for gradient nonlinearities during model fitting, and log files of EDDY processing. Furthermore, they provide 3.0T MRI data that structurally preprocessed for diffusion data with the HCP structural pipeline.

The DWI dataset were acquired using a 2D echo-planar imaging sequence with the following parameters: number of directions = 96, flip angle = 78°, repetition time/echo time = 5,200/89.5 ms, FOV = 1,584 mm × 1,848 mm, resolution = 1.25 mm isotropic, maximum *b*-value = 3,100 s/mm^2^, pixel bandwidth = 1,490 Hz/px, total acquisition time = 11 min 27 s. The T1-weighted MRI dataset for the localization of the seed voxels was acquired using a 3D MPRAGE sequence with the following parameters: repetition time/echo time = 2,400/2.14 ms, inversion time = 1,000 ms, flip angle = 8°, field-of-view = 22.4 cm × 22.4 cm, resolution = 0.7 mm isotropic, pixel bandwidth = 210 Hz/px, total acquisition time = 7 min 40 s.

The identification numbers of each 19 individuals selected from the HCP 3T-DWI dataset in this study are as follows: HCP102816, HCP104416, HCP105923, HCP108323, HCP109123, HCP111312, HCP111514, HCP114823, HCP115017, HCP115825, HCP116726, HCP118225, HCP125525, HCP126426, HCP128935, HCP130114, HCP130518, HCP131217, HCP131722. From these datasets, fully automated four-step data processing was undertaken in this study as follows: (1) Brain parcellation and track extraction; (2) Track validation and classification; (3) Track-density ratio mapping; and (4) Group data analysis.

### Brain Parcellation and Track Extraction

The anatomical T1 MR images in each dataset were parcellated individually using the Brainnetome atlas^1^, Fan et al., 2016 and FreeSurfer software^2^. The Brainnetome atlas is a highly detailedly parcellated framework of the human cortex based on the fiber connectivity model and Brodmann’s area (Brodmann, 1909; Fan et al., 2016). Supplementary Figure 1 and Supplementary Table 1 show the label number with the names and location of the parcellated Brainnetome brain areas.

After that, 5 million tracks were extracted per each individual MR-DWI data using the track extracting function in the MRtrix software (Brain Research Institute, Florey Neuroscience Institutes, Melbourne, Australia^3^). The fixed parameters for the entire dataset in this track extraction step were set as follows: tracking type = SD-PROB (probabilistic), direction of the fiber-tracking = uni-direction, step-size = 0.2 mm, curvature radius constraint = 0.8 mm, cutoff = 0.1. The inner scalp area of each MR brain image was used to set the seeding and masking regions in this track extraction procedure (Tournier et al., 2012; Cho et al., 2015b; Choi et al., 2019, 2020).

### Track Validation and Classification

The analysis and representation of the extracted track data were performed by homemade code with Matlab (MathWorks, Massachusetts, USA^4^), and the codes for all analyses in this report are available. As the first step, appropriate tracks in the obtained track dataset were selected by removing the noise tracks and ineffective tracks using the basic brain segmentation information by FreeSurfer (white matter area, cortical area, subcortical area). In other words, all extracted tracks have two end-terminals in each direction. We assessed and removed any track with a terminal in the white matter area as a noise track and any track without a terminal in the cerebral cortex as an ineffective track.

After that, the validated tracks were classified into four categories (tracks for projection, commissural, short association, and long association fiber) using the basic brain segmentation information that divided into three areas: cortical area in the RH and LH and subcortical area by FreeSurfer. In more detail, validated tracks with terminals in the subcortical structure were classified as projection fibers (subcortical←→cortical), and tracks that had cortical terminals in the contralateral hemisphere were classified as commissural fiber (cortical in LH←→cortical in RH). The remaining validated tracks that had cortical terminals in the ipsilateral hemisphere were classified as association fibers (cortical in LH←→cortical in LH, cortical in RH←→cortical in RH). The tracks for association fibers were classified once more into two categories by fiber length. Bajada et al. (2019) has characterized the short fiber as the fiber under 52 mm fiber length. In this study, a shorter than 6 cm track was classified as a short association fiber and a longer than 6 cm track as a long association fiber. Supplementary Figure 2 shows the actual tracks that are classified with this method. These distributed patterns of each classified fiber-tracking were well matched with the conventional anatomical knowledge about the fiber structure (Catani and Thiebaut de Schotten, 2008; Bajada et al., 2019).

### Track-Density Ratio Mapping

The categorized track dataset was used to obtain the track-density information in the cerebral cortex. We counted the number of terminals for each four categorized fiber per unit-sized voxel (1 mm^3^) area in all the cortical regions. The track-density value in each voxel is the number of terminals of each fiber type in the unit-sized voxel. The track-density ratio information was represented by density-dependent color intensity for each categorized fiber with three different primary colors (tracks for the commissural fiber = red, projection fiber = green, association fiber = blue) in each voxel.

**Figure 1:**
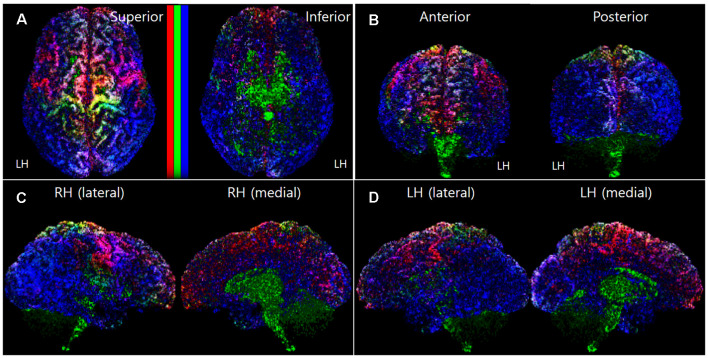
A representative voxel-based TDRM (dataset ID: 105923) in the cerebral cortex with the three different primary colors (tracks for commissural fiber = red, projection fiber = green, association fiber = blue). **(A)** Superior and inferior view. **(B)** Anterior and posterior view. **(C)** Right hemisphere in lateral and medial view. **(D)** Left hemisphere in lateral and medial view. LH, left hemisphere; RH, right hemisphere; TDRM, track-density ratio map.

To make the mapping result 3D-like-view from the 2D slices image dataset of the track-density information, we stacked each track-density slice image from the starting slice to the ending slice direction. For example, to get the 3D-like superior view of the brain mapping result, all the axial images of the mapping results were stacked from the inferior slice image to a superior slice image direction. In this slice stacking step, the noise data point and the black background data were removed in the 3D-like mapping result by excluding the voxel, which has a minimum track-density level. Figure 1 shows a representative result image (subject ID: 105923) of the track-density ratio map (TDRM) in the surface of the brain from various views.

**Figure 2:**
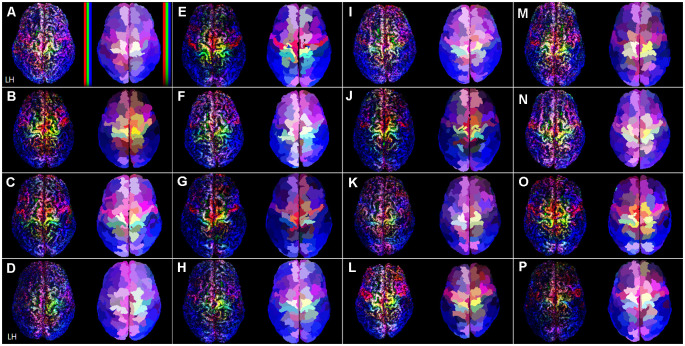
The TDRM from all 16 datasets in the superior view. In each image box, the left figures are the unit-sized voxel (1 mm^3^) based TDRMs, and the right figures are parcellation-piece-based TDRMs. In panel **(A)**, non-linear scaled color bars represent mapping density in each type of fiber track (tracks for commissural fiber = red, projection fiber = green, association fiber = blue). The capital letters in each image box **(A–P)** indicate the dataset ID (see Supplementary Table 2). TDRM, track-density ratio map.

**Figure 3:**
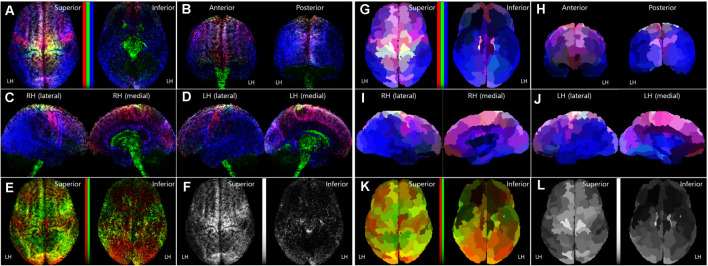
Normalized TRRMs from the 16 subject datasets. **(A–F)** Averaging image of the voxel-based TDRMs. **(G–L)** Parcellation-piece-based mean TDRMs (tracks_amount/piece_volume). The display scheme of each image **(A–D, G–J)** in this figure is the same as Figure 1. In panels **(A,E,F,G,K**), non-linear scaled color bars represent the mapping density of tracks for each fiber type. **(A–D, G–J)** TDRMs results with the three different primary colors (tracks for commissural fiber = red, projection fiber = green, association fiber = blue). **(E,K)** TDRMs for the association fiber-related tracks, with the two different primary colors (short association fiber = red, long association fiber = green). **(F,L)** Total track-density maps using the grayscaled intensity. LH, left hemisphere; RH, right hemisphere; TDRM, track-density ratio map.

The histogram of the track-density for each voxel in the TDRM showed the form of an exponential decay graph. To enhance the contrast of the mapping images, non-linear methods based on exponential functions were applied in the intensity mapping according to track density. In more detail, the mapping intensity according to track-density was determined by the logarithmic function based normalization, such as 1 − *e*^− 30 × *density/max_density*^ in the voxel-based ratio density mapping (Figures 1–3), 1 − *e*^− 8 × *density/max_density*^ in the parcellation-based ratio density mapping (Figures 2, 3), 1 − *e*^− 2 × *density/max_density*^ in the parcellation-based grayscaled total density mapping (Figures 3F,L). The column bars in Figure 1A, Figure 2A, and Figure 3 indicate the corresponding non-linear scale.

### Group Data Analysis

For the group data analysis, each validated track from 19 individual subjects were categorized by four kinds of fiber types. Supplementary Table 2 summarized the counting number of the validated tracks of each fiber type. Many tracks were terminated in the white matter area (not cortical area), and over 90% of the extracted tracks were excluded in the track validation step; the mean number of the validated tracks was 416,672 from 5 million extracted tracks. The data from individual ID 115825, 118225, and 130114 were excluded in the further analysis because of their anomalous high number of total validated tracks and abnormal track-ratio for commissural fibers compared with other datasets. Two kinds of group mapping were performed from the remaining 16 case datasets, voxel-based mean-TDRM in the surface area and parcellation-piece-based mean-TDRM.

To get the mean-TDRM in the surface of the normalized space by matching the voxel place across the subject brain, the fiber tracking results per each subject were transformed to Montreal Neurological Institute (MNI) template space through the “tcktransform” function in MRtrix. The transformed track per subject was reconstructed to the normalized TDRM, and the mean-TDRM in the surface was obtained by averaging the normalized TDRMs per voxel. The averaged mapping results help us to figure out the general trend of the individual TDRMs.

For quantitative group analysis, the mean-TDRM in the volume of the parcellated brain was composed and we used the Brainnetome-based parcellation information. Because the unit areas in this mapping approach are the piece_volume in the cortex, not a unit-sized single voxel on the surface, this mapping result is more reliable and reproducible than the prior voxel-based mapping result. Per each parcellation-piece, we measured the volume size of the parcellation-piece (piece_volume) and counted the number of the terminals (tracks_amount) of each categorized validated track. After that, we regarded the ratio “tracks_amount/piece_volume” of each parcellation-piece as the relative track-density in the parcellated brain area.

## Results

### Individual Data Observation

Figure 2 shows the superior view of the track-density ratio mapping results into the native space for all the 16 individuals’ data. The left and right images in each image box are the results of the voxel-based TDRM in the surface area and parcellation-piece-based TDRM in each volume. The voxel-based TDRMs that are on the left side are independent of the preceded brain parcellation information. Nevertheless, each mapping result shows a highly localized distribution according to the fiber type in the cerebral cortex. The major gyri and sulci boundaries, such as the longitudinal fissure and central sulcus, were clearly identified in the results. Furthermore, the mapping results in the superior brain show splendid colors cluster according to the types of fiber tracks.

On the other hand, the parcellation-piece-based TDRMs represented the track-density ratio in each cerebral cortex area based on the preceded brain parcellation information, the Brainnetome Atlas. A large proportion of the cerebral cortex is consistently covered with bluish color, which is the color assigned to tracks for association fibers, in both the voxel-based and parcellation-piece-based TDRMs. The group data about the tracks counting show that over 60% of the total validated tracks are association fibers (Supplementary Table 2). On the other hand, the tracking ratio of the projection fibers and commissural fibers was similar: 18.3% and 20.2%. The left images of each image box in Figure 2 reveal that the abundant commissural fibers (red) are located in the precentral gyrus and superior part of the frontal cortex. Meanwhile, the projection fibers (green) are dominant in the superior part of the postcentral gyrus. The yellowish color found in the superior part of the precentral gyrus in Figure 2 indicates that this region is densely packed with a similar proportion of commissural and projection fibers.

### Normalized Mapping of the Group Data

Figures 3A–F show the averaging image of the voxel-based TDRMs from the 16 subject datasets. Because of the track-normalization procedure with the MNI template brain, the mapping results are a little bit blurred. However, the averaging data’s mapping appearance is similar to the trend of the individual TDRMs’ aspect. For example, the difference of the track-density ratio between precentral gyrus for the motor control (red: commissural fiber) and postcentral gyrus for somatosensory (blue: association fiber) in the track-density ratio is clearly shown in the mean mapping result (Figure 3A) as well, like the individual mapping results (Figure 2).

Furthermore, the results in the medial view of the left and right hemispheres (Figures 3C,D) reveal that the major fiber in the subcortical area is the projection fiber (green), and the major fiber in the medial cortex except for the cingulate area (magenta) is the association fiber (blue) and commissural fiber (red). The result also shows that the high-density result for association fiber (green) in the temporal lobe and lateral part of the occipital lobe (Figure 3B).

### Quantitative Analysis of the Group Data

**Figure 4:**
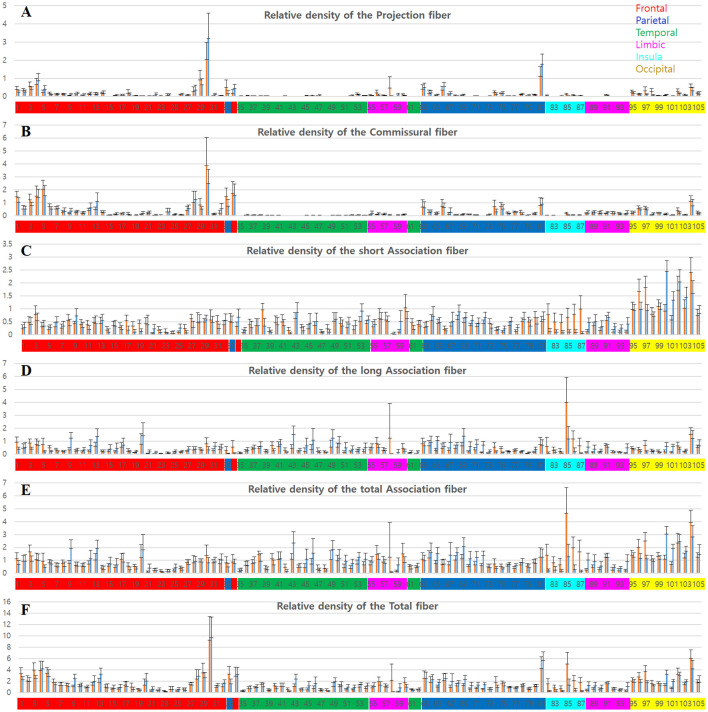
Graphs of the tracks density in each parcellation-piece from all 16 datasets per parcellation-piece. The X-axes indicate the parcellation label of the Brainnetome Atlas (see Supplementary Figure 1 and Supplementary Table 1). The label numbers were coded by color depending on the lobes of the brain as follows: frontal lobe (red), parietal lobe (blue), temporal lobe (green), limbic lobe (magenta), insular lobe (cyan), and occipital lobe (yellow). The Y-axes indicate the relative density of the tracks in the volume (tracks_amount/piece_volume). The bar in the left-side (red) and right-side (blue) in each label number is the results from the left and right hemispheres, respectively. **(A–E)** Mean and standard deviation of the relative track-density for the **(A)** projection, **(B)** commissural, **(C)** short association, **(D)** long association, **(E)** total association, and **(F)** total fibers.

The six graphs in Figure 4 show the relative track-density level (tracks_amount/piece_volume) in each parcellated brain area per each fiber category from the 16 validated datasets. In the graph of the tracks for projection fibers (Figure 4A), part of the superior frontal gyrus (1–5), precentral gyrus (28–30), superior parietal lobule (63, 66), and postcentral gyrus (81) are the regions with high track-density. The cortical distribution of the relative track-density of commissural fiber (Figure 4B) is similar to that of projection fiber in these figures. With the mentioned common regions, part of the middle frontal gyrus (12, 13), and paracentral lobule (33, 34) are additional high-density regions of the commissural fibers. The projection fibers have single terminals in the cerebral cortex, and the other is in the subcortical area. This is in contrast to commissural and association fibers, which have double terminals. Therefore, the level of the tracks for projection fibers density is about half of the tracks for commissural fiber, although their track-density was similar (Supplementary Table 2).

Meanwhile, the aspect of the distributions for the relative track-density of the association fiber (Figures 4C–E) was different. They were distributed widely throughout the cerebral cortex, except for some brain regions, such as the orbital gyrus (21–26). In the occipital cortex (95–105), tracks for short association fiber (Figure 4C) show relatively high-density (see Supplementary Figure 2), while tracks for long association fiber (Figure 4D) show relatively low-density (Bajada et al., 2019). In the result of the total track-density (Figure 4F), the most densely populated area of the nerve fiber in the brain was the precentral gyrus—trunk region (30). The superior frontal gyrus (1–7), paracentral lobule (33, 34), postcentral gyrus—trunk region (81) also had a high-density of the total nerve fiber in the graph.

The group data also show the asymmetricity of the track-density in the LH and RH. Although the total track-density had a similar level, there were significantly more tracks in the RH compared with the LH, in long association fibers (*p*-value = 0.0017). The mean of the relative track-density for each categorized track from 16 subject datasets in the LH and RH is as follows: projection fiber (LH: 0.1326, RH: 0.1399), commissural (LH: 0.2922, RH: 0.2672), short association (LH: 0.4804, RH: 0.4674), long association (LH: 0.3968, RH: 0.4255), total association (LH: 0.8772, RH: 0.8936), and total fiber (LH: 1.4345, RH: 1.4406). The density asymmetricity between hemispheres was particularly significant in association fibers. In the relative track-density for the total association fiber (Figure 4E), the entorhinal cortex (58, *p*-value = 0.02) and insular gyrus (82–87, *p*-value = 2.52e-08) showed significant LH dominance, and the temporal gyrus (41–54, *p*-value = 5.46e-06) and parietal lobule (63–73, *p*-value = 1.07e-06) showed significant RH dominance.

### Quantitative Mapping of the Group Data

The parcellation-piece-based track-density data in Figure 4 were reconstructed in the parcellated brain (ID: 105923) to make the data more understandable. The images in Figures 3G–L show the TDRMs based on the mean track-density level of the 16 datasets in each parcellated region. These figures more clearly reveal that the association fiber is dominant in the temporal, inferior parietal, and lateral occipital lobes (bluish color in the inferior and posterior part of the cerebral cortex). They also indicate that the abundant projection and commissural fibers are in the superior part of the frontal and parietal cortex (un-bluish color in the anterior and superior part of the cerebral cortex). Because this quantitative mapping analysis assessed the cerebral cortex alone, the track-density for projection fibers observed in the diencephalon, cerebellum, and diencephalon in Figures 3C,D with green color is not reflected in this map. This is also why the tracks for the greenish projection fibers are less prominent in this parcellation-piece-based TDRM.

Figures 3K,L include one or two-dimensional track-density information in the parcellated brain area from superior (left) and inferior (right) views. Figure 3K with Figures 4C,D shows that the track distribution for the short association fibers (red) is denser than the long association fibers (blue) in broad regions in the inferior frontal gyrus of the LH, and occipital lobe. Also, Figure 3L implies that the superior frontal cortex has the most density of nerve fibers among all the brain regions, and the inferior frontal, and inferior frontal, and inferior temporal areas have sparser nerve fibers than other brain regions.

## Discussion and Conclusions

The TDRMs presented here were implemented using a fully automated protocol; therefore, the reproducibility is expected of the results according to the described method. However, there is a flaw in the approach due to the problem in the DWI-MR image. The inferior part of the brain, such as the orbitofrontal cortex and medial temporal gyrus, are likely to be distorted in DWI-MRI images due to susceptibility artifacts. Because of the artifacts, it is hard to avoid distortion of the TDRM data in the inferior part of the brain. The signal loss due to distortions in the DWI data should be considered in interpreting the track-density data in inferior brain regions. Other distortions in the extracted track are the bias to terminate preferentially on gyral crowns rather than the banks of sulci, and inaccessibility to the cortical surface in the current fiber tracking algorithms (Reveley et al., 2015; Schilling et al., 2018). Because of the inaccessibility, 90% of the extracted tracks have been excluded in the track validation step from this study. Furthermore, because of the gyral bias, the signal loss in the sulci area should be considered in interpreting the track-density maps as well.

Finally, the TDRM results are affected by the fiber extraction method. The whole-brain random fiber tracking approach that we adopt as a conventional default fiber extraction method is robust in human bias or mistake. However, it does not mean that the conventional method is optimal, therefore, it can introduce biases in detecting fiber pathways and limiting coverage. These problems can be solved by improving the scheme of fiber extraction. For example, underestimated commissural fiber mass in the parietal lobe in our results can be enhanced by adapting the multi-stage region-of-interest-based fiber tracking (Jarbo et al., 2012).

The TDRMs results can be discussed in relation to the cortical circuit as they can be affected by the regional specialization of the cortical connectivity. The cortical circuit information based on the cytoarchitecture scale analysis differentiates between input and output fiber information to the cortex and reflects the connectivity within the cortical area (Elston, 2003; Rockland, 2019), which cannot be reflected in the TDRMs results that are based on the macro or mesoscale analysis. Several reports describe that the cortical circuit in the cerebral cortex is not generalized and has regional differences, through the observation of the size or dendritic property in the supragranular pyramidal cells (Jacobs et al., 2001; Luebke, 2017). Moreover, they suggested these structural differences with the cortical difference in the integrated ability or functional capacities because these differences are significant in the high integration area, especially in the prefrontal cortex that is considered the high-level cognitive functional area (Elston et al., 2001; Jacobs et al., 2001; Elston, 2003; Luebke, 2017). The cortical specialization results have a chance to be correlated and combined with our track-density results. Actually, there is a macro-scaled connectivity study based on the tract tracer injection in the cortex (Markov et al., 2014; Oligschläger et al., 2019), and the projection of each fiber type (projection, commissural, and association fibers) to the cortex is distinguished at the laminar level (Figure 2 in Rockland, 2019). For example, like cortical specialization results, the TDRM in the frontal area is distinguished from the TDRM in other posterior areas that showing association fiber dominance. Furthermore, like the connection at the laminar level (Figure 1 in Rockland, 2019), ascending sensory area is clearly distinguished from the descending motor area, in the TDRM.

In the proposed TDRM method, the density information of multiple categorized fibers in the cerebral cortex was represented in a single image using a combination of the three different primary colors. The representation approach using the combination of the primary colors for the track-density imaging is already implemented in diffusion tensor imaging (DTI) data (Pajevic and Pierpaoli, 1999) and track-density imaging (TDI) data (Calamante et al., 2010). The difference is that the three primary colors in the DTI and TDI reflect fiber direction, not fiber type. Like the DTI and TDI images do, even by a single resulted image, the TDRMs can represent the multiple types of track-density more effectively and aggregately. Furthermore, the mapping results are more comfortable to figure out the overall state of the multiple types of track-density.

Because of the strength, the mapping method used in this study can be applied to various fields. For example, this method is applicable to brain function studies. The functional role of each categorized nerve fiber is likely to differ based on differences in their trajectories. For example, the projection fiber is intimately involved in the arousal response and motor control, and damage to association fiber can result in altered language function and behavior (Afifi and Bergman, 1998; David and Anil, 2010). Therefore, analyses of track-density and fiber-type in each cortical region and their regional difference allow us to get insight for understanding each cortical region’s functional specialization in brain computations. For example, TDRMs of the group data (Figure 3) indicate that roughly the posterior part of the brain has a high distribution density of association fibers, while the anterior part of the brain is shown a high distribution density of projection and commissural fibers. This difference in the fiber-type between anterior to posterior parts of the brain can be correlated with that the overall functional role between them is discriminated, such as motor-related and sensory-related function, respectively.

Furthermore, functional localization between the left and right hemispheres may be interpreted by analyzing the asymmetry in the TDRM. From Figures 3G–L and Figure 4, we found several brain areas that show the significant asymmetry in track-density, particularly in the track for association fibers. The asymmetry in the fiber structure between the left and right hemispheres suggests the functional laterality in the corresponding area. Finally, this technique has the potential for diagnosing brain diseases by characterizing the anatomical distinction of the fiber amount and fiber type between populations in the cerebral cortex (Nir et al., 2015; Henderson et al., 2020). The TDRM hopes to be used as an alternative biomarker to diagnose neurodegenerative diseases (Prasad et al., 2013; Nir et al., 2015) or tumor (Stadlbauer et al., 2010; Henderson et al., 2020) in the brain associated with abnormalities in the distribution characteristics of that.

In conclusion, we implemented TDRMs in this study, a novel cortical mapping method based on the nerve fiber’s type using MR tractography data. Their quantitative results from the group data analysis provided more detailed and reliable information on the track-density ratio, including the hemispheric asymmetries in the association fibers’ density. We expect that the information from the TDRMs provides new dimensional information for the understanding of brain anatomy and function, and neurodegenerative diseases.

## Data Availability Statement

The original contributions presented in the study are included in the article/Supplementary Material, further inquiries can be directed to the corresponding author/s.

## Author Contributions

S-HC suggested the model, processed the data, and drafted the manuscript. GJ and Y-EH processed the HCP data. Y-BK assisted with the neuroanatomical information. Z-HC and HL performed the super-resolution tractography and its application to the study of neural circuits. All authors contributed to the article and approved the submitted version.

## Conflict of Interest

The authors declare that the research was conducted in the absence of any commercial or financial relationships that could be construed as a potential conflict of interest.

## Publisher’s Note

All claims expressed in this article are solely those of the authors and do not necessarily represent those of their affiliated organizations, or those of the publisher, the editors and the reviewers. Any product that may be evaluated in this article, or claim that may be made by its manufacturer, is not guaranteed or endorsed by the publisher.

## Footnotes

^1^ http://atlas.brainnetome.org

^2^ https://surfer.nmr.mgh.harvard.edu/

^3^ https://mrtrix.readthedocs.io/en/latest/

^4^ https://mathworks.com/
