# Supplementary material for: Track-Density Ratio Mapping With Fiber Types in the Cerebral Cortex Using Diffusion-Weighted MRI
Source: Front Neuroanat. 2021 Jul 23;15:715571. doi: 10.3389/fnana.2021.715571 (PMC8441551; doi:10.3389/fnana.2021.715571)
Supplement: Supplementary file 1 [file Data_Sheet_1.docx]

Supplementary Material

# Supplementary Figures and Tables

## Supplementary Figures

**Supplementary Figure S1.** Parcellation scheme of the human brain in the Brainnetome Atlas. (A) lateral view, (B) medial view, (C) ventral view. Fan L, Li H, Zhuo J, Zhang Y, Wang J, Chen L, Yang Z, Chu C, Xie S, Laird AR, Fox PT, Eickhoff SB, Yu C, Jiang T (2016) The Human Brainnetome Atlas: A New Brain Atlas Based on Connectional Architecture. Cereb Cortex 26:3508-3526.


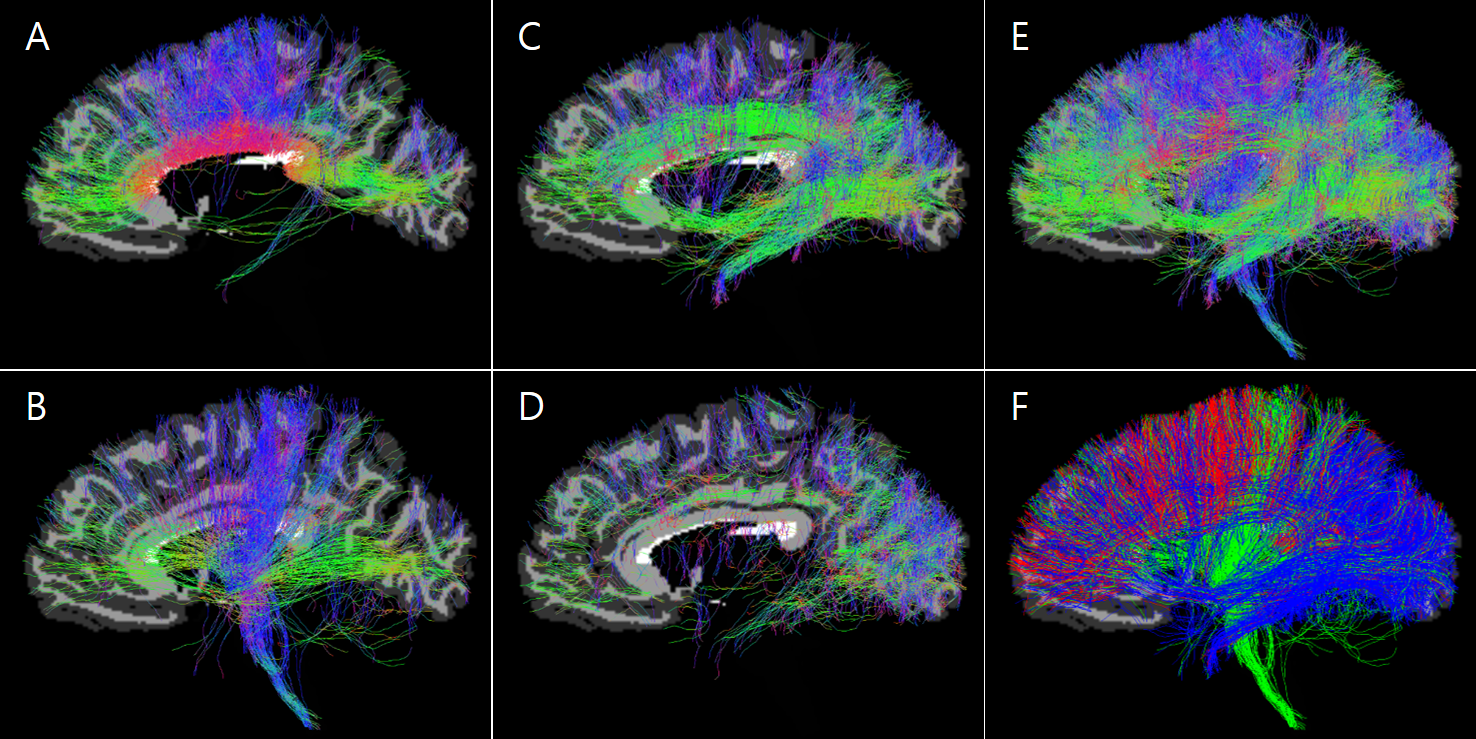


**Supplementary Figure S2.** Sagittal view of the tracks validation and classification result from the 50000 extracted tracks from a representative dataset (ID: 105923). (A-D) Tracks classified as tracks for (A) commissural fibers (n = 820), (B) projection fibers (n = 750), (C) long association fibers (n = 1057), and (D) short association fibers (n = 1449). (E-F) All validated fiber tracks (n = 4076) as (E) directional colors and (F) labeled color by fiber types (tracks for commissural fiber = red, projection fiber = green, association fiber = blue).

## Supplementary Tables

**Supplementary Table S1.** The summarized list of the 105 parcellated brain areas in the Brainnetome atlas. The summarized list of the label number and anatomical and cyto-architectonic descriptions and location and the MNI coordination of the 105 parcellated brain areas in the Brainnetome atlas.

|  | **Subject ID** | Projection | Commissural | Short | Long | **Total** |
| --- | --- | --- | --- | --- | --- | --- |
| **A** | **102816** | 77350 | 89460 | 182207 | 130053 | 479070 |
| **B** | **104416** | 84552 | 106135 | 130020 | 97323 | 418030 |
| **C** | **105923** | 74136 | 85653 | 141395 | 107022 | 408206 |
| **D** | **108323** | 79852 | 79259 | 172014 | 115401 | 446526 |
| **E** | **109123** | 74085 | 65200 | 130450 | 100775 | 370510 |
| **F** | **111312** | 79130 | 71669 | 148097 | 132017 | 430913 |
| **G** | **111514** | 55905 | 64355 | 121411 | 109078 | 350749 |
| **H** | **114823** | 76710 | 66661 | 175019 | 109963 | 428353 |
| **I** | **115017** | 72837 | 81679 | 170765 | 101909 | 427190 |
|  | **115825** | 138550 | 110349 | 257062 | 123415 | 629376 |
| **J** | **116726** | 65710 | 68427 | 110363 | 93640 | 338140 |
|  | **118225** | 54718 | 47724 | 154461 | 93261 | 350164 |
| **K** | **125525** | 86599 | 106202 | 149627 | 145154 | 487582 |
| **L** | **126426** | 89593 | 108314 | 127692 | 111130 | 436729 |
| **M** | **128935** | 78220 | 91075 | 149355 | 97091 | 415741 |
|  | **130114** | 50107 | 149586 | 138603 | 121012 | 459308 |
| **N** | **130518** | 83506 | 92616 | 160554 | 101184 | 437860 |
| **O** | **131217** | 79029 | 82431 | 127023 | 101107 | 389590 |
| **P** | **131722** | 65428 | 86573 | 129234 | 120329 | 401564 |
|  | **mean count(16)** | 76415.1 | 84106.8 | 145326.6 | 110823.5 | 416672.1 |
|  | **mean ratio(16)** | 18.3 | 20.2 | 34.9 | 26.6 | 100 |

**Supplementary Table S2.** The counting number of the validated tracks in the 4 categories (projection, commissural, short and long association fibers) per each dataset ID. Five million extracted tracks were processed per each dataset. The capital Arabic letters in the first column indicate the dataset ID in Fig. 3. Three datasets (ID 115825, 118225, 130114) were excluded by their abnormality; therefore, their data were not regarded in the mean track count and mean track ratios in this Table.
